# Maintaining accuracy and expanding access: evaluating the efficacy of the Botucatu Abbreviated Breast MRI Protocol

**DOI:** 10.61622/rbgo/2024rbgo55

**Published:** 2024-07-26

**Authors:** Eduardo Carvalho Pessoa, Thais Paiva Moares, Heverton Leal Ernesto de Amorim, Henrique Lima Couto, Joelcio Francisco Abbade, Suzana Shinomia, Carla Priscila Kamiya Carvalho Pessoa, Eliana Aguiar Petri Nahas

**Affiliations:** 1 Department of Gynecology and Obstetrics Universidade Estadual Paulista “Júlio de Mesquita Filho” Botucatu SP Brazil Department of Gynecology and Obstetrics, Universidade Estadual Paulista “Júlio de Mesquita Filho”, Botucatu, SP, Brazil.; 2 Rede Mater Dei de Saúde e Redimama Belo Horizonte MG Brazil Rede Mater Dei de Saúde e Redimama, Belo Horizonte, MG, Brazil.; 3 Redimama Belo Horizonte MG Brazil Redimama, Belo Horizonte, MG, Brazil.; 4 Ud Diagnóstico por Imagem João Pessoa PB Brasil Ud Diagnóstico por Imagem, João Pessoa, PB, Brasil.; 5 Department of Radiology Botucatu Medical School Universidade Estadual Paulista “Júlio de Mesquita Filho” Botucatu SP Brazil Department of Radiology, Botucatu Medical School, Universidade Estadual Paulista “Júlio de Mesquita Filho”, Botucatu, SP, Brazil.

**Keywords:** Magnetic resonance imaging, Botucatu Abbreviated Protocol, Breast neoplasms, Screening

## Abstract

**Objective:**

Our study evaluated the effectiveness of the Botucatu Abbreviated Protocol in breast magnetic resonance imaging (MRI) within Brazil’s public healthcare system, focusing on its impact on patient access to MRI exams.

**Methods:**

This retrospective study involved 197 breast MRI exams of female patients over 18 years with histological breast carcinoma diagnosis, conducted at Hospital das Clínicas de Botucatu - UNESP between 2014 and 2018. Two experienced examiners prospectively and blindly analyzed the exams using an Integrated Picture Archiving and Communication System (PACS). They first evaluated the Botucatu Abbreviated Protocol, created from sequences of the complete protocol (PC), and after an average interval of 30 days, they reassessed the same 197 exams with the complete protocol. Dynamic and morphological characteristics of lesions were assessed according to BI-RADS 5th edition criteria. The study also analyzed the average number of monthly exams before and after the implementation of Botucatu Abbreviated Protocol.

**Results:**

The Botucatu Abbreviated Protocol showed high sensitivity (99% and 96%) and specificity (90.9% and 96%). There was a significant increase in the average monthly MRI exams from 6.62 to 23.8 post-implementation.

**Conclusion:**

The Botucatu Abbreviated Protocol proved effective in maintaining diagnostic accuracy and improving accessibility to breast MRI exams, particularly in the public healthcare setting.

## Introduction

Breast cancer is the most prevalent cancer among women globally, with an estimated 3 million new cases and 1 million deaths projected by 2040.^(1)^ In 2020, it was the leading cause of cancer-related mortality in women across over 100 countries, with a rate of 13.6 per 100,000 inhabitants.^(2)^ The mechanisms of carcinogenesis in this heterogeneous disease are not fully understood, presenting challenges for primary prevention.^(3)^Early detection, especially through mammographic screening, has been shown to reduce breast cancer mortality by 16-40%.^(3,4)^ However, mammography’s sensitivity is lower in cases of dense breasts and rapidly growing tumors, which are common in high-risk women, resulting in less than 50% sensitivity in these scenarios.^(5,6)^ Unlike mammography and breast ultrasonography, breast Magnetic Resonance Imaging (MRI) is a functional technique, introduced by Heywang et al.^(7)^ and Kaiser and Zeitler in the 1980s.^(8)^ It assesses the permeability of blood vessels using an intravenous contrast agent (gadolinium), leading to higher signal intensity on T1-weighted images. The greater the neoangiogenesis, the more leaky vessels there are, allowing for quicker flow and extravasation of contrast agents, resulting in rapid local enhancement.^(9)^ Therefore, breast MRI has higher sensitivity and specificity compared to conventional examinations (mammography and ultrasonography), is not affected by breast density, and is highly sensitive in situations of greater biological aggressiveness and rapid growth. It is the preferred screening method for high-risk individuals, offering superior performance over mammography and ultrasonography, including significantly lower rates of interval cancer, a recognized indicator of mortality.^(10-13)^Nevertheless, the cost and tolerability of the examination are major barriers to accessing breast MRI, particularly in screening scenarios. Regarding cost, recent studies have shown that breast MRI can be cost-effective in the long term, even for women with intermediate risk due to high breast density. Meanwhile, tolerability greatly improves with the adoption of shorter protocols, which also help reduce costs.^(10,14,15)^

In 2014, Christiane Kuhl^(15)^ introduced the Abbreviated Breast MRI Protocol (AB-MRI) to enhance accessibility by reducing examination duration, increasing tolerability, and thereby decreasing costs. The initial protocol involved a pre-contrast acquisition and a post-contrast acquisition, including the calculation of a post-contrast subtracted image and a maximum intensity projection image.^(10,15)^ Since then, numerous centers have adopted abbreviated protocols, and recent studies suggest that the sensitivity and specificity of AB-MRI are comparable to longer protocols. However, the lack of a standard breast MRI protocol, both for abbreviated and full-scale protocols, makes general assessment challenging, as each approach leads to different diagnostic accuracies, with lingering concerns about the standardization and validation of abbreviated protocols.^(5,16-18)^ Our study aims to evaluate the efficacy of the Botucatu Abbreviated Protocol (BAP) and its impact on access to breast MRI in the context of Brazil’s public health service.

## Methods

This is a retrospective review of 197 breast MRI examinations of female patients over 18 years with a histological diagnosis of breast carcinoma, conducted during the diagnostic investigation process. The study also examined the number of monthly exams conducted with the complete protocol and the number since the implementation of the BAP. All patients were treated at the Hospital das Clínicas de Botucatu - UNESP, between 2014 and 2018, and the study was approved by the institution’s ethics committee.

Exams Two independent reviewers with over 10 years of experience in breast imaging and MRI were invited. The reviewers used an Integrated Picture Archiving and Communication System (PACS) with dedicated breast imaging monitors for exam interpretation. The readers were blinded to the patients’ clinical history, including the reason for the exam, patient risk, diagnosis, and previous imaging studies. They first evaluated the BAP, followed by the complete protocol (PC), after an average interval of 30 days. For the BAP exams, patients were numbered sequentially from 1 to 197 and in reverse order for the PC from 197 to 1. The dynamic and morphological characteristics of all detected lesions were analyzed for both breast MRI protocols according to the criteria of the American College of Radiology Breast Imaging Reporting and Data System (BI-RADS) 5th edition lexicon.^(19)^

Breast MRI exams were performed using a 3T system (Siemens) with a dedicated surface breast coil. Patients were positioned prone with both breasts hanging freely in the bilateral surface coil, thus avoiding any compression during the diagnostic procedure.

Complete Protocol (PC) - Duration 27 minutes and 22 seconds

The Siemens 3T machine was used, with the patient in prone position using a dedicated breast coil. Standard protocol: 3 mm slices, with a minimum matrix of 256 x 256.

1. Localization sequence - 00:25 min.
2. Axial STIR sequence - 03:25 min.
3. Axial T2 BLADE sequence - 04:21 min.
4. Right sagittal T2 SPAIR sequence - 02:08 min.
5. Left sagittal T2 SPAIR sequence - 02:08 min.
6. Diffusion sequence - 04:07 min.
7. Contrast phase: gadolinium contrast used, dosage of 0.1nmol/kg, speed of 3ml/s.
  **-**Axial T1 - one T1 axial sequence without contrast and five contrast-enhanced axial T1 sequences, total time 07:25 min.
  **-**Late axial T1 with fat saturation - 03:23 min.
8. Post-processing, post-contrast subtraction sequences, and MIP subtraction data.

Botucatu Abbreviated Protocol - 9 minutes and 28 seconds Based on sequences from the standard protocol, an abbreviated protocol was created consisting of:

1. Localization sequence - 00:25 min.
2. Axial T2 W sequence lasting 02:48 min.
3. One axial T1 sequence without contrast and four contrast-enhanced axial T1 sequences, total time 6:15 min. Gadolinium contrast used, dosage of 0.1nmol/kg, speed of 3ml/s.
4. Post-processing, post-contrast subtraction sequences, and MIP subtraction data.

We conducted statistical analysis to examine the variation in the number of monthly breast MRI examinations before and after the implementation of the BAP. We employed independent t-tests to compare the monthly examination means in these two periods, aiming to quantify the impact of BAP on the hospital’s MRI service efficiency.

The difference between sequences and radiologists’ evaluations was analyzed using generalized estimating equations with robust sandwich variance estimates. A p-value of <0.05 was adopted for statistical significance. All analyses were performed using Stata/SE 12.0 for Windows.

The National Research Ethics approved the protocol under number 4.456.897 (CAAE: 39816020.0.0000.5411).

## Results

In our sample of 197 reviewed cases, it was found that 35% of patients were under 50 years old. The majority (95.4%) were diagnosed with invasive carcinoma. Regarding immunohistochemical characteristics, estrogen receptors were positive in 72% of tumors, while progesterone receptors were positive in 60.3% of cases. HER2 positivity was identified in 27.4% of carcinomas. Furthermore, the KI67 proliferation index exceeded 20% in 53% of tumors. Table 1 details patient distribution by age, histological diagnosis, and immunohistochemical characteristics.

**Table 1:** Description of patients’ age and anatomopathological characteristics of the lesions

Variables	n(%)
Age	
Less than	29(14.6)
40 40-49 years	40(20.4)
More than 50 years	128(65.0)
Histological Type	
Iinvasive ductal carcinoma (IDC)	170(86.3)
Lobular carcinoma (LC)	11(5.6)
Ductal carcinoma in situ (DCIS)	7(3.5)
PAGET (DCIS)	2(1.1)
Others	7(3.5)
Tumor Grade	
1	6(3)
2	84(42.6)
3	88(44.7)
No information	19(9.7)
Estrogen Receptor (ER)	
ER Positive	142(72.0)
ER Negative	51(25.9)
NO Information	4(2.1)
PR Positive	119(60.3)
PR Negative	70(35.5)
NO Information	8(2.2)
HER 2	
Positive	54(27.4)
Negative	134(68.0)
Doubtful or No Information	9(4.6)
KI67	78(39.6)
UP to 20	105(53.3)
Greater than 20 NO Information	14(7.1) 197(100)
Total	

Invasive ductal carcinoma (IDC), Lobular carcinoma (LC), Ductal carcinoma in situ (DCIS), Estrogen Receptor (ER), Progestrone Receptor (PR)

The average lesion size measured by MRI was 2.8 cm for examiner 1 and 2.95 cm for examiner 2. Most lesions were classified as masses by both assessors. Regarding baseline enhancement, examiner 1 classified most exams as moderate or intense (60.8% in PAB, 71.8% in PC), while examiner 2 identified most as minimal or slight (57.3% in BAP, 57.5% in PC). Nodule shape and margin were predominantly irregular and non-circumscribed in both protocols, and the uptake pattern was heterogeneous/annular in over 80% of cases, regardless of the protocol. Over 97% of findings were categorized as suspicious (BI-RADS 4 or 5). Details of MRI findings are presented in table 2.

**Table 2:** Description of lesions according to examiners using BI-RADS lexicon

Characteristics of the lesions (BI-RADS)	Examiner 2		Examiner 1	
	Abbreviated	Full	Abbreviated	Full
Tumor size average	2.8 cm	2.8 cm	3.0 cm	2.9cm
Mass lesion	85.7%	84.7%	88.8%	90.3%
Non-mass lesion	14.3%	15.3%	11.2%	9.7%
Minimal/discreet baseline enhancement	39.2%	28.2%	57.3%	57.5%
Moderate/accentuated baseline enhancement	60.8%	71.8%	42.7%	42.5%
Oval/round nodule	33.9%	48%	5.6%	8.4%
Irregular nodule	66.1%	52%	94.4%	91.6%
Circumscribed margin	14.2%	25.7%	5.6%	2.3%
Non-circumscribed margin	85.8%	74.3%	94.4%	97.7%
Homogeneous uptake	19.2%	15.5%	13.8%	10.4%
Heterogeneous/annular uptake	80.8%	84.5%	86.2%	89.6%
BI-RADS category 4 or 5	97.9%	97.9%	98.4%	97.9%
BI-RADS category 1, 2, or 3	2.1%	2.1%	1.6%	2.1%

Tables 3 and 4 display sensitivity, specificity, positive predictive value (PPV), negative predictive value (NPV), and accuracy for each examiner in the evaluated protocols. Both showed high sensitivity, with 99% for examiner 1 and 96% for examiner 2. Specificity was 92.5% in PC and 90.9% in BAP for examiner 1, and 96.9% in BAP and 95.5% in PC for examiner 2. PPV and NPV were also high: examiner 1 achieved 91.9% PPV in BAP and 92.5% in PC, while examiner 2 had 97.0% in BAP and 95.5% in PC. Accuracy was 95% in PAB and 95.9% in PC for examiner 1, and 96.4% in BAP and 95.7% in PC for examiner 2. Table 5 details undiagnosed cases, including specific case losses in both protocols by both examiners.

**Table 3:** Sensitivity, specificity, positive predictive value, negative predictive value, and accuracy of examiner 1 in the abbreviated and complete protocols

		Breast cancer		Total
		Present	Absent	
BAP	Suspect	193	17	210
	Not suspect	2	170	172
	Total	195	187	382
Sensitivity = 99.0% (96.3% - 99.7%)				
Specificity = 90.9% (85.9% - 94.2%)				
Positive Predictive Value (PPV) = 91.9% (87.4% - 94.8%)				
Negative Predictive Value (NPV) = 98.8% (95.9% - 99.7%)				
Accuracy = 95.0% (92.4% - 96.8%)				
		Present	Absent	Total
FULL	Suspect	197	14	211
	Not suspect	2	173	175
	Total	199	187	386
Sensitivity = 99.0% (96.4% - 99.7%)				
Specificity = 92.5% (87.8% - 95.5%)				
Positive Predictive Value (PPV) = 93.4% (89.2% - 96.0%)				
Negative Predictive Value (NPV) = 98.9% (95.9% - 99.7%)				
Accuracy = 95.9% (93.4% - 97.4%)				

**Table 4:** Sensitivity, specificity, positive predictive value, negative predictive value, and accuracy of examiner 2 in the abbreviated and complete protocols

		Breast cancer		Total
		Present	Absent	
BAP	Suspect	191	6	197
	Not suspect	8	185	193
	Total	199	191	390
Sensitivity = 96.0% (92.3% - 97.9%)				
Specificity = 96.9% (93.3% - 98.5%)				
Positive Predictive Value (PPV) = 97.0% (93.5% - 98.6%)				
Negative Predictive Value (NPV = 95.9% (92.0% - 97.9%)				
Accuracy = 96.4% (94.1% - 97.8%)				
		Present	Absent	Total
Completo	Suspect	193	9	202
	Not suspect	8	183	191
	Total	201	192	393
Sensitivity = 96.0% (92.3% - 98.0%)				
Specificity = 95.3% (91.3% - 97.5%)				
Positive Predictive Value (PPV) = 95.5% (91.8% - 97.6%)				
Negative Predictive Value (NPV = 95.8% (91.6% - 97.9%)				
Accuracy = 95.7% (93.2% - 97.4%)				

**Table 5:** Correlation of cases lost by examiners in different protocols with pathological findings

	histology	Lesion size (cm)	Estrogen receptor	Progesterone receptor	HER 2	Ki67(%)
Lost case BAP						
Examiner 1						
Number 54	IDC	3.0	Positive	Positive	Negative	20
Number 50	Paget-DCIS	0.6	Positive	Positive	Negative	15
Number 28	IDC	0.9	Positive	Positive	Negative	8
Number10	Paget-DCIS	0.8	Positive	Positive	Negative	30
Examiner 2			Positive	Positive		
Number 79	IDC	1.1	Positive	Positive	Negative	5
number 50	Paget-DCIS	0.6	Positive	Positive	Negative	15
number 10	Paget-DCIS	0.8	Positive	Positive	Negative	30
Lost case Full						
Examiner 1						
number 50	Paget-DCIS	0.6	Positive	Positive	Negative	15
number 30	IDC	1.5	Positive	Positive	Negative	5
number 28	IDC	1.0	Positive	Positive	Negative	8
number 10	Paget-DCIS	0.8	Positive	Positive	Negative	30
Examiner 2			Positive	Positive		
number 76	ILC	3.5	Positive	Positive	Negative	5
number 50	Paget-DCIS	0.6	Positive	Positive	Negative	15
number 10	Paget-DCIS	0.8	Positive	Positive	Negative	30

Comparative analysis of the monthly frequency of breast MRI exams revealed a significant increase after the introduction of the BAP. Before BAP implementation (2013-2018), the monthly average was 6.62 exams. This average increased to 23.8 monthly exams after adopting BAP, representing a statistically significant increase (p < 0.003135). Chart 1 shows the monthly frequency of exams conducted in each year and the average monthly exams per year.

**Chart 1:** The number of breast MRI examinations performed each year and month, along with the average monthly exams for each year

Year	January	February	March	April	May	June	July	August	September	october	november	december	average monthly
2014	5	3	4	10	8	7	9	8	8	6	8	6	6.8
2015	1	4	6	6	8	4	7	8	7	5	7	1	5.3
2016	7	7	8	4	4	2	8	7	4	9	3	7	5.8
2017	5	7	6	8	8	10	9	9	7	8	8	0	7
2018	9	6	8	12	7	9	9	10	8	7	8	6	8.2
2019	7	10	12	10	17	24	19	26	24	17	24	18	17.3
2020	14	24	22	9	12	19	26	21	10	14	17	21	17.4
2021	17	32	26	15	24	28	9	34	27	31	26	28	24.7
2022	29	11	38	40	51	34	38	44	36	40	37	35	36

## Discussion

Since Christiane Kuhl’s 2014 publication,^(15)^ numerous studies on AB-MRI have emerged, including systematic reviews/meta-analyses and a prospective multicenter clinical trial (Eastern Cooperative Oncology Group–American College of Radiology Imaging Network [ECOG-ACRIN] 1141 [EA1141]). AB-MRI, featuring protocols under 10 minutes, has been integrated into clinical workflows in various organizations. However, clinical implementation poses challenges such as validation and operational execution.^(16-18,20)^Our group developed the BAP within the public health system. BAP, as demonstrated in our research and supported by meta-analyses, offers a safe and effective alternative to longer protocols.^(16,17)^It showed high sensitivity and specificity, comparable to full protocols and other abbreviated protocols in literature, utilizing a T2-weighted sequence (targeting specificity, peritumoral, and axillary regions) and four post-contrast T1 phases (preserving kinetic data and enhancing sensitivity for less angiogenic tumors), with sensitivity rates were 99% MRI is considered a multiparametric technique, wherein T2-weighted images and a dynamic T1-weighted contrast-enhanced sequence form the basis of a standard protocol. T1 images are typically acquired in the axial plane, which is faster than sagittal acquisition and provides a better overall view of both breasts.^(9)^This phase of the exam begins with a T1-weighted sequence acquired before the administration of contrast, followed by post-contrast acquisitions. For contrast injection, the recommended dose of gadolinium should not exceed the maximum of 0.1 mmol per kilogram of body weight, as there is no evidence that higher doses yield better results. The injection should be done using an automatic injector at a flow rate of 2 to 3 mL/sec, and the contrast bolus should be followed by saline (approximately 20 mL).^(9,21,22)^This contrast phase is crucial for the detection of breast cancer, as most lesions will enhance 60-90 seconds after the injection of gadolinium, making it essential to obtain an image during this period. In this contrast phase, images created with fat subtraction in T1 greatly facilitate diagnosis as they help highlight structures that enhance contrast. Thus, for lesion detection, the acquisition of two T1-weighted images at specified time points (one before and another approximately 90 seconds after contrast administration) is usually sufficient, as demonstrated in studies of abbreviated protocols for breast MRI.^(9)^All other sequences improve the differentiation of breast lesions, aiming to prevent false-positive and false-negative classifications. When analyzing the BAP there is one pre-contrast T1-weighted sequence and four post-contrast T1 sequences, maintaining all kinetic characteristics of a standard protocol to prevent false positives and negatives. These results are in line with Baxter’s findings, reinforcing the efficacy of BAP.^(16)^

Magnetic Resonance Imaging is currently the most sensitive imaging technique for detecting breast cancer, applicable in screening, staging, and evaluating therapeutic response. Recent years have seen growing awareness of breast MRI’s benefits, particularly in screening high-risk patients (lifetime risk > 20%).^(23)^In Brazil, three medical societies recently reinforced MRI’s role in early breast cancer detection in high-risk patients and those with dense breasts.^(24)^ However, breast MRI is often criticized for its high cost, especially in screening. Addressing this debate, recent cost-effectiveness studies have shown that breast MRI is economically viable in both dense breast screening and high-risk (lifetime risk > 20%) scenarios.^(25,26)^ In Brazil’s distinct private and public health systems, there is a rising interest in making breast MRI available to high-risk populations. Particularly in developing countries like Brazil, efforts are necessary to enhance access, reduce costs, and improve patient management. The development of Abbreviated Breast MRI (AB-MRI) as a cost-reducing and access-increasing solution in both public and private settings has been significant.^(27)^ The implementation of the BAP greatly enhanced the efficiency of MRI services within the Unified Health System (SUS), shortening examination times and increasing the monthly average of exams from 6.62 to 23.8, thus expanding access and tolerability. Currently, BAP allows comprehensive screening of all high-risk women within SUS, in line with literature emphasizing the benefits of breast MRI optimization, including cost reduction and improved tolerability.^(25-29)^A recent systematic review also supports MRI’s cost-effectiveness for screening high-risk populations, typically with limited access in public health systems.^(30)^

We recognize the limitations of our study, which include a retrospective cohort of breast carcinoma cases and the abbreviated protocol derived from the complete protocol of a single center. Nevertheless, all cases were prospectively evaluated by two experienced breast imaging specialists, particularly in breast MRI. The examiners were blinded to the patients’ medical histories and their respective diagnoses.

The adoption of this method can bring significant benefits, including cost reduction and improved access to diagnostics, in both public health systems and health insurance environments. However, it is important to note that the results obtained in our research may not be universally applicable across different centers. We acknowledge that the concept of an abbreviated MRI protocol for breast cancer screening is not novel in the scientific publication landscape. Nonetheless, we emphasize the importance and specific value of the BAP within the Brazilian context, contributing to expanding access to breast MRI and enhancing early detection of breast cancer in high-risk women in our country. Thus, the BAP can promote more inclusive and accessible screening for all population layers, contributing to a more equitable approach especially in the public health environment.

## Conclusion

Our study highlighted the efficacy of the Botucatu Abbreviated Protocol in detecting breast carcinomas, demonstrating performance comparable to the standard protocol.
